# A Quick Guide to Large-Scale Genomic Data Mining

**DOI:** 10.1371/journal.pcbi.1000779

**Published:** 2010-05-27

**Authors:** Curtis Huttenhower, Oliver Hofmann

**Affiliations:** Department of Biostatistics, Harvard School of Public Health, Boston, Massachusetts, United States of America; Whitehead Institute, United States of America

## Introduction

For the first several hundred years of research in cellular biology, the main bottleneck to scientific progress was data collection. Our newfound data-richness, however, has shifted this bottleneck from collection to analysis [1]. While a variety of options exists for examining any one experimental dataset, we are still discovering what new biological questions can be answered by mining thousands of genomic datasets in tandem, potentially spanning different molecular activities, technological platforms, and model organisms. As an analogy, consider the difference between searching one document for a keyword and executing an online search. While the tasks are conceptually similar, they require vastly different underlying methodologies, and they have correspondingly large differences in their potentials for knowledge discovery.

Large-scale genomic data mining is thus the process of using many (potentially diverse) datasets, often from public repositories, to address a specific biological question. Statistical meta-analyses are an excellent example, in which many experimental results are examined in order to lend statistical power to a hypothesis test (e.g., for differential expression) [2], [3]. As the amount of available genomic data grows, however, exploratory methods allowing hypothesis generation are also becoming more prevalent. The ArrayExpress Gene Expression Atlas, for example, allows users to examine hundreds of experimental factors across thousands of independent experimental results [4]. In most cases, though, an investigator with a specific question in mind must collect relevant data to bring to bear on a question of interest. Some examples might be:

- If you've obtained a gene set of interest, in which tissues or cell lines are they coexpressed?
- If you assay a particular cellular environment, are there other experimental conditions that incur a similar genomic response?
- If you have high-specificity, low-throughput data for a few genes, with what other genes do they interact or coexpress in high-throughput data repositories? Under what experimental conditions, or in which tissues?

Bringing large quantities of genomic data to bear on such questions involves three main tasks: establishing methodology for efficiently querying large data collections; assembling data from appropriate repositories; and integrating information from a variety of experimental data types. Since the technical [5]–[7] and methodological [8]–[10] challenges in heterogeneous data integration have been discussed elsewhere, this introduction will focus mainly on the first two points. As discussed below, the computational requirements for processing thousands of whole-genome datasets in a reasonable amount of time must be addressed, either algorithmically or using cloud or distributed computing [11], [12]. Subsequently, data collection is sometimes easy—as is increasingly the case for high-throughput sequencing, individual experiments can themselves be the sources of large data repositories. In other cases, a biological investigation might benefit from the inclusion of substantial external or public data.

## Methods and Pitfalls in Manipulating Genomic Data

A point that must be emphasized when dealing with very large genomic data collections is that many convenient computational tools for individual dataset analysis will scale poorly to repositories of hundreds or thousands of genome-scale experimental results. Scripting environments such as R/Bioconductor [13] and MATLAB (The MathWorks) should be used with caution to avoid excessive runtimes. Similarly, data storage can be as great or greater a concern as data processing: plain text or XML storage formats, while conveniently human-readable, can waste unsustainable amounts of space for large repositories.

Solutions to these technical issues include software and data access methodologies specifically tailored to large-scale data manipulation. Three broad categories of solutions exist: Web applications that aggregate information from multiple sources, programmatic APIs that allow sophisticated computational queries of individual large data sources, and do-it-yourself solutions that rely on manually obtaining and processing bulk data from public repositories. In the first category, most current bioinformatic systems include online interfaces, but these generally provide analyses of individual datasets rather than large compendia. Notable exceptions include the STRING [14] and BioMart [15] tools, which aggregate a large number of functional and sequence annotation data sources, respectively. Integrated results and data portals are also available for many model organisms, including HEFalMp [16], Endeavour [17], and the Prioritizer [18] for human data, integrated within- [19] and across-species [20] results for *Caenorhabditis elegans*, bioPIXIE [21] and SPELL [22] for *Saccharomyces cerevisiae*, and a variety of tools for other systems [23]–[25].

While these online tools provide pre-computed data mining results, a second option is to perform tailored queries of experimental results from one or more large public repositories. This adds a level of complexity, since you must still decide on appropriate downstream analyses of the retrieved data, but the heavy lifting of data normalization, filtering, and search is still done by the remote system. Manual portals to such information are the core of canonical interfaces at the National Center for Biotechnology Information [26] and European Bioinformatics Institute [27], and workflow systems such as Taverna [28] and Galaxy [29] are emerging to automate significant portions of these analysis pipelines. Most major data repositories now offer programmable interfaces using one of several common protocols: HTTP (i.e., programmatic URLs or REST) [26], [27], SOAP [30], [31], or bioinformatic services such as DAS [7], BioMOBY [32], or Gaggle [33]. These protocols provide a way to pose sophisticated queries to a data repository, leaving you to examine only the end products of interest.

The greatest level of flexibility in large-scale biological data mining is offered by manually processing bulk experimental data, which of course also incurs the greatest level of time commitment and overhead. However, this is currently one of the only ways in which sophisticated multifactorial queries can be executed. If you're interested in identifying potential targets of yeast cell cycle kinases under a variety of culture growth conditions, even a relatively complex large-scale computational screen will likely be simpler than running new corresponding high-throughput assays:

1. By examining the *S. cerevisiae* Gene Ontology (GO) [34] annotations at the *Saccharomyces* Genome Database [35], we find that the intersection between the *cell cycle* process (669 genes) and the *protein kinase activity* function (135 genes, both terms downloadable at AmiGO [36]) yields a list of 51 genes.
2. By downloading the DIP [37], MINT [38], and bioGRID [39] interaction databases (discussed below) in bulk and searching for all interactions in which these genes' products participate, we obtain 7,830 potential kinase-target pairs.
3. By downloading all Gene Expression Omnibus (GEO) [40] yeast expression data in bulk (also discussed below), calculating all normalized correlations using Sleipnir ([11], a calculation taking <1h), and listing only correlations stringently significant at a corrected 0.01 level (*p* = 1.2×10^−5^, z = 4.22), we find 81 cell cycle kinase-target pairs with high correlation under some experimental condition.
4. It is vital to evaluate the accuracy of our predictions, although since GO was used as part of the input data, care must be taken to avoid a circular evaluation. In this case, the non-kinase interaction partners were predicted solely based on experimental interactions and coexpression, and we find that 45 of them (∼25%, hypergeometric *p*<10^−8^) indeed have known roles in the cell cycle.

**Figure 1:**
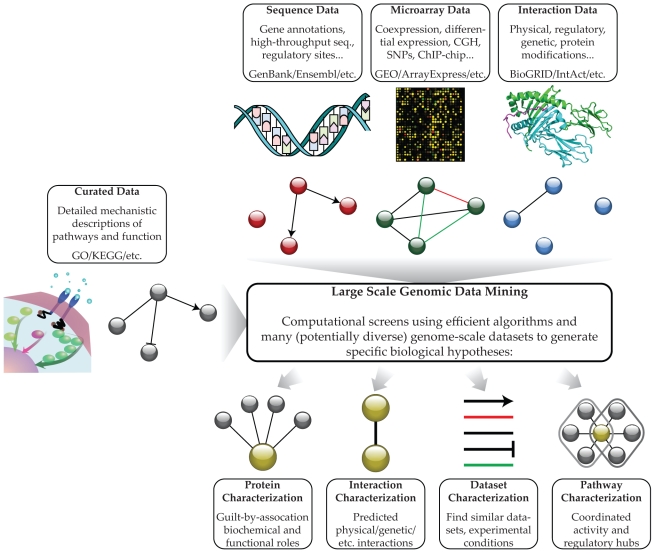
Large-scale genomic data mining. A schematic overview of possible inputs, data sources, network models, and output predictions from computational screens leveraging many genome-scale datasets. Note that both the “output” pathway model and the “input” experimental data are represented as networks: directed regulatory binding site targets, undirected weighted coexpression, and undirected interactions, respectively. As demonstrated by the sample analysis in Box 1, biological networks provide a uniform framework within which both experimental data and predicted models can be represented, facilitating integrative analyses.

Note that in each of these steps, experimental data of several different types is processed using a uniform network model, and this workflow for large-scale biological data analysis is summarized in Figure 1; a description of the analysis is provided in Box 1 and detailed commands are listed in Text S1. This small example is obviously biologically somewhat naive, but it demonstrates the remarkably nuanced questions that can be answered using large-scale data mining even without complex machine learning methodology.

**Box 1.** An example using multiple genome-scale data repositories to determine potential kinase-target interactions active during the *S. cerevisiae* cell cycle.

For step-by-step instructions on performing each task, please see Text S1.

1. Retrieve lists of known yeast cell cycle and protein kinase genes from the Gene Ontology [34] using the AmiGO [36] Web service.
2. Intersect these two gene sets to find protein kinases potentially involved in the cell cycle.
3. Retrieve lists of experimentally determined protein–protein interactions from the DIP [37], MINT [38], and bioGRID [39] databases.
4. Map all appropriate gene identifiers to gene symbols using information from BioMart [15].
5. Taking the union of these three databases, identify any pairs of interacting proteins in which at least one partner is a member of the cell cycle protein kinase list. Note that this will provide a conservative underestimate, since many transient kinase–target interactions are difficult to detect based on high-throughput data.
6. Retrieve yeast expression data from GEO [40] and convert each dataset into a normalized coexpression network using the Sleipnir software [11].
7. Extract all gene pairs correlated above a multiple hypothesis corrected 0.01 significance level, and intersect these pairs with the list of cell cycle protein kinase interactions.
8. This produces a list of potential cell cycle-linked phosphorylation targets that is based on protein kinases known to be involved in the cell cycle, interacting with the putative target, and coexpressing strongly with it under some experimental condition.
9. Finally, evaluate the proposed list's plausibility by examining how many of the non-kinase partners are known cell cycle genes.

Unsurprisingly, a number of common technical pitfalls arise in large-scale data analysis. Even structured databases can break down in the face of thousands of whole-genome interactomes, leading most current large-scale data repositories to employ some combination of file system-based flat file storage archives and binary formats (including GenBank's ASN.1 PER [26], BioHDF [41], and Sleipnir's DAB [11]). Data transfer mechanisms for bulk data are often limited to FTP or Aspera (http://www.asperasoft.com), although experimental metadata is often available through sophisticated programmable interfaces [40], [42], [43]. Several reviews have been written dealing with inter-study data normalization [8], [44], particularly for microarrays [45]–[47]—although perhaps the simplest yet most important normalizations required are often chromosomal coordinates and gene, transcript, and protein identification schemes [48].

## Genomic Data Resources

Three practical impediments to large-scale integrative data mining are data availability, data size, and algorithms and models for integration. As discussed above, the challenges inherent in manipulating large data can often be overcome through compact encodings and awareness of efficiency issues. Similarly, although many sophisticated systems for biological data integration exist [8]–[10], [49], they are not always necessary in order to discover new biology in large data collections. As demonstrated by the toy analysis above, simply asking the right questions of several different data repositories can rapidly generate novel biological hypotheses. It remains to discover and catalog the availability and scope of these repositories; the annual *Nucleic Acids Research* database issue [50] is an excellent resource for this, as are online database aggregators (e.g., [51]–[53] and http://biodatabase.org), and several primary biological data types and sources are presented here in summary.

### High-Throughput Sequencing

Next-generation short-read DNA sequencing is rapidly becoming a current-generation technology and producing ever-longer read lengths. While the purpose of this manuscript is not to address the (serious) informatic requirements needed for processing raw sequence data, several points raised by [1] are worth summarizing. Current sequencers can generate up to 400 million 50–100-bp reads per run, and this number will be obsolete soon after this manuscript is published. Performing even the simplest analyses on this data, let alone assembly, polymorphism detection, annotation, or other complex tasks, requires sophisticated computational hardware *and* software. Few cookie-cutter solutions are available, given how rapidly the technology continues to change, but online forums such as SEQanswers (http://seqanswers.com) are currently one of the best resources for up-to-date information on short-read sequencing.

When investigating individual organisms' genomes (discussed below in more detail), many of the tools for large-scale sequence mining are focused on the study of variation: across disease state, tissue, or pathogen samples (e.g., The Cancer Genome Atlas [54] and the Cancer Genome Project [55]), structurally or polymorphically across individuals (e.g., the 1,000 Genomes Project [56] and the Personal Genome Project [57]), or phylogenetically across species (e.g., Genome 10K [58]). Particularly for phylogeny and evolutionary relationships, a variety of tools are available online that efficiently summarize very large sequence collections; EMBOSS [59], MEGA [60], MEGAN [61], and mothur [62] are only a few of the creatively named systems available in this area.

An interesting large-scale data mining opportunity afforded by modern sequencing techniques is provided by metagenomic repositories such as CAMERA [63], MG-RAST [64], and IMG/M [65], all of which offer tools for inter-study comparisons of multiple environmental or microfloral datasets. For instance, an experimenter can easily upload an entire metagenome to MG-RAST and receive a detailed profile of the community's metabolic potential; using CAMERA, fragment recruitment profiles can be generated comparing any pair of metagenomes. Simultaneously considering the functional diversity of a metagenome, its constituent organisms, and the associated experimental metadata allows a single analysis to scale from molecular mechanisms to global ecology [66].

### Whole-Genome Sequences

The first widely used large-scale biological data repositories were (arguably) for reads deposited during the Human Genome Project and other pioneering sequencing projects, and these remain important sources of annotated genomic sequences. GenBank [67] has diversified to include a variety of online and offline tools such as the Genome Workbench, and Ensembl [68] provides an invaluable online window into a number of genome builds. The Sanger Institute hosts a number of additional genome resources (http://www.sanger.ac.uk/Projects/), and the Joint Genome Institute provides several microbial genomes and associated tools [69]. Sequence annotations have been reviewed elsewhere [70] and include everything from open reading frames through regulatory sites to chromatin structure and epigenetics; much of this information is available through a uniform interface at the UCSC Genome Browser [71]. Sequence data have been highly standardized over the years, with most raw sequences provided as FASTA or its variants, detailed annotations provided as GenBank/EMBL files, and brief annotations as GFFs. Most sequence manipulation software will recognize all of these formats [72].

### Microarrays

Similarly, gene expression microarrays were the first functional data to be analyzed on a large scale, although applications of high-throughput sequencing are poised to overtake them in widespread data availability. The GEO [40] and ArrayExpress [42] databases are the most common sources of array data, with Celsius [73], field-specific resources such as Oncomine [74], and institute-specific databases [75] providing additional datasets. Both GEO and ArrayExpress provide programmatic interfaces and structured FTP file systems for bulk analysis. GEO data are standardized around the SOFT text file format [40] and ArrayExpress around the MGED MAGE format family [76]; both are variants of tab-delimited text and can be manipulated by a variety of publicly available tools [77], [78] or custom software.

### Physical, Genetic, and Regulatory Interactomes

Interactomes are significantly more diverse than sequence and expression data, both in their biological grounding and their electronic availability and distribution. For a subset of the many available physical, genetic, and regulatory interaction databases, we refer the reader to previous articles in the *PLoS Computational Biology* Getting Started series [79]. These data are distributed in a range of formats and with a variety of experimental metadata. The fundamental computational data being communicated is most often an unweighted (possibly directed) graph, and interactome data thus lends itself well to large-scale exploration using simple Boolean operations and graph mining algorithms [80], [81]. More biologically focused investigation can be done using, for example, PSI-formatted files containing experimental and biological metadata [82].

### Other Genomic Data Types and Sources

This is only a small selection of the data resources that can be mined integratively to address biological questions, with structural [83], [84], proteomic [85], [86], and metabolic [87] databases being obvious large-scale omissions. A final data type that must be considered, however, is not directly experimental; curated pathway and structured knowledge resources are invaluable in the planning and validation of large-scale data mining [34], [88]–[90]. Two vital considerations when using such resources are, first, that they are originally based on published literature and experimental results. Subtle issues of circularity can arise when curated resources are used to supplement or validate data mining results, since the data being analyzed may itself have contributed to the curation process. Second, we have as yet to discover and catalog all biological knowledge—when used as gold standards, even the best-curated resources can be incomplete in the face of the billions of datapoints now being generated by the field on a regular basis, with important consequences in computational learning and evaluation [91].

## Outlook

With almost every type of biological data accumulating at an exponential rate, large-scale genomic data mining is increasingly becoming a necessity. For computational investigators, this represents a clear opportunity for methodology development; since data are becoming available at a rate that outpaces even Moore's law, it is not enough to wait for faster computers to execute longer and longer queries, and new bioinformatic tools must be developed with an eye to scalability and efficiency (e.g., through massive parallelization). However, the opportunity for biological investigation is at least as large. Nature has already harnessed scalability to her own advantage, and the combinatorics of the genetic code, multimodal and combinatorial regulation, cellular differentiation, and temporal development ensure that even our current wealth of data provide an incomplete view of biological complexity. A simple justification for broad-ranging computational screens of genomic data is their speed and low cost as a precursor to more extensive laboratory work. An even more compelling motivation, though, is the fact that the extent and complexity of biological systems may best be discovered by simultaneously considering a wide range of genome-scale data.

## Supporting Information

Text S1 An example using multiple genome-scale data repositories to determine potential kinase-target interactions active during the *S. cerevisiae *cell cycle.
(0.07 MB DOC)
