# Supplementary material for: A Quick Guide to Large-Scale Genomic Data Mining
Source: PLoS Comput Biol. 2010 May 27;6(5):e1000779. doi: 10.1371/journal.pcbi.1000779 (PMC2877728; doi:10.1371/journal.pcbi.1000779)
Supplement: Text S1 — An example using multiple genome-scale data repositories to determine potential kinase-target interactions active during the S. cerevisiae cell cycle. (0.07 MB DOC) [file pcbi.1000779.s001.doc]

An example using multiple genome-scale data repositories to determine potential kinase-target interactions active during the *S. cerevisiae* cell cycle.

1. Navigate to AmiGO at <http://amigo.geneontology.org/>
2. In the search box, type "cell cycle" and click Submit Query. Click the first result, "cell cycle".
3. Scroll down; in the list of species, select "Saccharomyces cerevisiae" and click Set Filters.
4. Above the Term Information header, a link will read "### gene product associations →", currently 668. Click this link.
5. Click the link to download all associations in "gene association format" and/or right-click and choose "Save As." Call this file cell_cycle.txt.
6. Repeat steps 2-5 using "protein kinase activity" as the query. Call this file kinase_activity.txt.
7. At a Linux or Cygwin ([http://www.cygwin.com](http://www.cygwin.com/)) command prompt, type the following command to create a file cell_cycle_genes.txt listing cell cycle genes:

cut -f3 cell_cycle.txt | sort | uniq > cell_cycle_genes.txt

1. Repeat step 7 using the input kinase_activity.txt to create the output kinase_activity_genes.txt.
2. Create a list of potential cell_cycle_kinases.txt using the following command:

sort -m cell_cycle_genes.txt kinase_activity_genes.txt | uniq -d > cell_cycle_kinases.txt

1. You can count the number of genes in this list using the command:

wc -l cell_cycle_kinases.txt

1. Obtain a mapping from UniProt IDs to yeast gene symbols by going to [http://www.biomart.org](http://www.biomart.org/).
2. Click Martview. Select the "Ensembl 56 Genes" database (the default) and, subsequently, "Saccharomyces cerevisiae genes".
3. Click Attributes, then expand the + beside GENE. Uncheck "Ensembl Gene ID" and "Ensembl Transcript ID". Check "Associated Gene Name".
4. Expand the + beside EXTERNAL. Check "UniProt/SwissProt Accession".
5. Click Results. Check "Unique results only" and click Go. The resulting file will be saved as mart_export.txt.
6. Download the DIP Saccharomyces-specific plain text file (currently Scere20081014.txt) from [http://dip.doe-mbi.ucla.edu](http://dip.doe-mbi.ucla.edu/). After logging in, this file is found under the Files/Species link in "MI tab" format.
7. Create a list of interacting UniProt gene pairs output to dip_uniprot.txt using the following command:

cut -f1-2 Scere20081014.txt |
ruby -e 'STDIN.each do |s|
 if( s =~ /uniprotkb:(\w+).+uniprotkb:(\w+)/ );
 puts( "#{$1}\t#{$2}" ); end; end' |
sort | uniq > dip_uniprot.txt

1. Map these UniProt identifiers to Saccharomyces genes in the file dip.txt using the command:

ruby -e 'h = {};
IO.foreach( "mart_export.txt" ) do |s|
 a = s.strip.split( /\t/ ); h[a[1]] = a[0]; end;
STDIN.each do |s|
 a = s.strip.split( /\t/ );
 if( ( t1 = h[a[0]] ) && ( t2 = h[a[1]] ) );
 if( t2 < t1 ); t2,t1 = t1,t2; end;
 puts( [t1, t2].join( "\t" ) ); end; end' <
dip_uniprot.txt | sort | uniq > dip.txt

1. Download the MINT Saccharomyces-specific plan text file (currently 2009-11-05-mint-Saccharomyces.txt) from [http://mint.bio.uniroma2.it](http://mint.bio.uniroma2.it/). This file is found under the Download link in "MINT flat file" format.
2. Create a list of interacting gene pairs output to mint.txt using the following command:

grep '4932..*4932' 2009-11-05-mint-Saccharomyces.txt | cut -f6 |
ruby -e 'STDIN.each do |s|
 a = s.strip.split( /;/ );
 a.length.times do |i|
 t1 = a[i]; ((i+1)...a.length).each do |j|
 t2 = a[j]; puts( [[t1, t2].min, [t1, t2].max].join( "\t" ) );
 end; end; end' |
sort | uniq > mint.txt

1. Download the bioGRID Saccharomyces-specific plain text file (currently BIOGRID-ORGANISM-Saccharomyces_cerevisiae-2.0.59.tab.txt) from [http://www.thebiogrid.org](http://www.thebiogrid.org/). This file is found under the downloads link under "Current Release", contained inside the BIOGRID-ORGANISM-2.0.59.tab.zip archive.
2. Create a list of interacting gene pairs output to biogrid.txt using the following command:

cut -f3-4 BIOGRID-ORGANISM-Saccharomyces_cerevisiae-2.0.59.tab.txt |
ruby -e 'STDIN.each do |s|
 a = s.strip.split( /\t/ );
 if( a.length != 2 ); next; end;
 if( a[1] < a[0] ); a.reverse!; end;
 puts( a.join( "\t" ) ); end' > biogrid.txt

1. Create a list of potential kinase_interactions.txt using the following command:

cat dip.txt mint.txt biogrid.txt | sort | uniq |
ruby -e 'h = {};
IO.foreach( "cell_cycle_kinases.txt" ) do |s|
 h[s.strip] = true; end;
STDIN.each do |s|
 a = s.strip.split( /\t/ );
 if( h[a[0]] || h[a[1]] ); puts( s ); end; end' > kinase_interactions.txt

1. You can count the number of interactions in this list using the command:

wc -l kinase_interactions.txt

1. Retrieve a list of GEO datasets gds.txt for *Saccharomyces cerevisiae* using the NCBI E-Utils as detailed at <http://www.ncbi.nlm.nih.gov/geo>.

wget -O - 'http://eutils.ncbi.nlm.nih.gov
 /entrez/eutils/esearch.fcgi?db=gds&term=yeast[orgn]&retmax=5000&usehistory=y' |
ruby -e 'strQK = strWE = nil;
STDIN.each do |s|
 if( s =~ /QueryKey\>([^<]+)\</ ); strQK = $1; end;
 if( s =~ /WebEnv\>([^<]+)\</ ); strWE = $1; end; end;
 system( "wget -O - \"http://eutils.ncbi.nlm.nih.gov" +
 "/entrez/eutils/efetch.fcgi?db=gds&mode=file&report=docsum&" +
 "query_key=#{strQK}&WebEnv=#{strWE}\"" )' |
ruby -e 'STDIN.each do |s|
 if( s =~ /^\d+:\s+(GDS\d+)/ );
 puts( $1 ); end; end' > gds.txt

1. Download the SOFT files corresponding to these datasets:

ruby -e 'STDIN.each do |s|
 system( "wget ftp://ftp.ncbi.nih.gov/pub/geo/DATA/SOFT/GDS/#{s.strip}.soft.gz" );
end' < gds.txt

1. Unzip the SOFT files:

gunzip *.soft.gz

1. Convert the SOFT files into PCLs (<http://smd.stanford.edu/help/formats.shtml>):

ruby -e 'ARGV.each do |s|
 s =~ /(\S+)\.soft/;
 File.open( $1 + ".pcl", "w" ) do |f|
 IO.foreach( s ) do |t|
 if( t =~ /^[!^#]/ ); next; end;
 a = t.strip.gsub( "null", "" ).split( /\t/ );
 if( a[0] == "ID_REF" );
 f.puts( "GID\tNAME\tGWEIGHT\t" + a[2, a.length].join( "\t" ) );
 elsif( a[0] != "EMPTY" );
 f.puts( [a[1], a[0], 1].concat(
 a[2, a.length] ).join( "\t" ) ); end; end; end; end' *.soft

1. Download and build the Sleipnir software from <http://function.princeton.edu/sleipnir>. In particular, you will need the Distancer and Dat2Dab tools; external libraries will not be necessary.
2. Calculate all GEO datasets' normalized correlation scores using the command:

ruby -e 'ARGV.each do |s|
 s =~ /(.*)\.pcl/;
 system( "Distancer -o #{$1}.dab < #{s}" ); end' *.pcl

1. Create a list of highly correlated gene pairs correlations.txt using the command below. Note that the z-score cutoff of 4.22 corresponds to a genome-wide Bonferroni-corrected p-value of 0.01.

ruby -e 'ARGV.each do |s|
 system( "Dat2Dab -m -i #{s} -c 4.22" ); end' *.dab |
ruby -pe 'a = $_.strip.split( /\t/ );
 a = a[0,2];
 if( a[1] < a[0] ); a.reverse!; end;
 $_ = a.join( "\t" ) + "\n"' | sort | uniq > correlations.txt

1. Create a list of coexpressed kinase/target gene pairs kinase_correlations.txt using the command:

sort -m kinase_interactions.txt correlations.txt | uniq -d >
kinase_correlations.txt

1. These pairs can be counted using the command:

wc -l kinase_correlations.txt

1. Finally, we will evaluate these predictions by counting the non-kinase interaction partners known to be involved in the cell cycle. Create a list of all genes in the kinase/target pairs:

sed 's/\t/\n/' kinase_correlations.txt | sort | uniq > kinase_correlation_genes.txt

1. Identify the predicted cell cycle kinase targets by excluding the input protein kinases from this list:

cat kinase_correlation_genes.txt kinase_activity_genes.txt | sort |
uniq -u > predicted_kinase_targets.txt

1. Last, we can identify how many of these predicted targets are known cell cycle genes occurring in our original cell cycle list (which was not directly used to predict these targets):

cat predicted_kinase_targets.txt cell_cycle_genes.txt | sort | uniq -d |
wc -l

1. To test the significance of this overlap, consider four values: the total number of genes in which we looked for interactions (i.e. any yeast gene occurring in the input interaction databases), the set of cell cycle genes, the set of non-kinase interaction targets, and the size of the intersection calculated above. In current data, these four numbers are 6,553, 669, 174, and 45, respectively. The significance of the 45-gene overlap given this input can be tested using the cumulative hypergeometric distribution:

$$P(hits\geq45)=\sum_{i=45}^{174} \frac{\left(\begin{matrix} 669 \\ i \end{matrix}\right)\left(\begin{matrix} 6553-669 \\ 174-i \end{matrix}\right)}{\left(\begin{matrix} 6553 \\ 174 \end{matrix}\right)}\approx2\cdot10^{-9}$$

The value can be calculated using implementations provided online (e.g. [http://www.nr.com](http://www.nr.com/)) or interactively using tools such as <http://stattrek.com/Tables/Hypergeometric.aspx> or <http://easycalculation.com/statistics/hypergeometric-distribution.php>.
